# The Relations of Bacteria to Disease

**Published:** 1892-10

**Authors:** Charles M. Blackford

**Affiliations:** Lynchburg, Va.


ATLANTA
### MEDICAL AND SURGICAL JOURNAL.

VOL. IX. OCTOBER, 1892. NO. 8.

EDITED BY
LUTHER B. GRANDY, M. D., AND MILLER B. HUTCHINS, M. D.,
WITH THE CO-OPERATION OF
H. V. M. MILLER, M. D., LL. D., VIRGIL O. HARDON, M. D., AND
FLOYD W. McRAE, M. D.

### *ORIGINAL COMMUNICATIONS.*

### THE RELATIONS OF BACTERIA TO DISEASE.*

BY CHARLES M. BLACKFORD, M. D.,
LYNCHBURG, VA.

The term *bacteria* was originally applied to a single class of micro-organisms, and strictly it still is so used, but, in ordinary usage, it is generic, and will be used as such in this paper. The effect of these bacterial studies is well summed up by Senn, in the introduction to his work on "Surgical Bacteriology" thus: "During the last fifteen years there has been more real advance made in surgical pathology than during twenty centuries preceding them. Bacteriology opened a new era for surgical pathology. The knowledge which has accumulated from the bacteriological investigation of diseases has opened new fields of usefulness for the surgeon. Many diseases hitherto treated uselessly with drugs by the physician are now successfully treated by surgical measures. At this time surgical pathology has almost become synonymous with surgical

* Read at the meeting of the Medical Society of Virginia, September, 1892.

bacteriology." Such being the case, it is highly important that something be known by every one of the well established facts of this subject.

With this in view, it will not be out of place to consider the various ways in which the bacteria affect a higher organization. These ways may be roughly divided into two classes, one in which the bacterium directly attacks and destroys the tissue in which it has obtained lodgment, and another, in which a by-product of the life of the micro-organism is the agent that immediately does the damage. Thus in inflammation followed by pus formation, the pyogenic bacteria are probably directly responsible, but in tetanus the active poison is an alkaloidal product, or mixture of such products, resulting from the life of the bacillus tetani. These alkaloidal products may be isolated, and are as certain to produce tetanus if introduced in sufficient quantity into the system as morphia is to produce its physiological effects.

But in addition to the bacteria that are always pathogenic, there are others that are only so at times. In other words, some bacteria which normally are not capable of causing disease may, under peculiar environment, acquire malignancy and be pathogenic. Conversely, some bacteria that are violently malignant may, by cultivation in an unsuitable medium, become attenuated, so that on inoculation a very mild type of the disease will follow, sometimes only a local manifestation. There is reason to think that the "comma" bacillus of Asiatic cholera exhibits these variations of malignancy. The environment producing these variations may be the soil in which the micro-organisms are grown, the temperature, the presence or absence of oxygen, and some conditions as yet unknown. The diet on which the subject of the experiment has subsisted has a marked influence. In the Bradford epidemic of anthrax this was shown by a curious fact. It was noticed that the majority of the cases among the wool-sorters occurred on Sunday, or between Saturday and Monday. This was holiday time, as the mills were closed during that period, and much curiosity was aroused to discover what excited the dormant bacilli. It had been found that herbivorous animals were peculiarly liable to attacks of this disease, and that it was difficult, if not impossible, to produce it in carnivorous animals. Investigation showed that the wool-sorters ate largely of vegetables during their brief holiday, and thus rendered themselves an easier prey to the disease. The bacillus of mouse septicæmia is rapidly fatal to the house mouse, but is harmless to the field mouse. Here we have two animals of the same species responding to the same agent differently because of their different diet. Similarly rats vary in susceptibility as they are fed on vegetable or animal diet. Some bacteria seem to depend on the secretions of other varieties for malignancy; as, united they are malignant, divided they are harmless. These varieties greatly complicate the study of bacteriology, and impede our progress toward a complete comprehension of the subject.

Before considering the separate bacteria, it may be well to state briefly the modes by which the different species are differently situated. These are (1) direct microscopical examination, (2) the appearance of the "culture" in the tubes or other receptacle, (3) the behavior of the bacteria or their products with chemical reagents, and (4) the results of inoculations practiced on animals. The first of these, direct examination, is, when unaided by any of the others, of relatively little importance, as but few varieties possess such morphological peculiarities as to render them recognizable. Of course it makes out the distinction between the cocci and the bacilli, the one appearing as minute dots or spheres, the other as little rods. The cocci in turn can be divided into the streptococci, which appear as rows of little dots, the diplococci, which are in pairs, and the staphylococci, which are clustered into groups. The bacilli give less scope for morphological classification. Some are curved, as is the comma bacillus; some are short, with clubbed ends, as is the bacillus typhosus; some bear a spore on one end, as the bacillus tetani; some are motile, as is the bacillus of relapsing fever, but on the whole mere inspection would enable one to differentiate but few species.

The appearance of the culture is somewhat more satisfactory; indeed some varieties have received names from the appearance of their cultures. They may not liquefy the medium in which the growth takes place; the growth may be visible on some soils and invisible on others, as the bacillus typhosus, which is visible in gelatine but not so on potato. In some cases in which the active agent causing a disease has not been made out, it has at least been settled that it does not belong to a certain one of these classes. Thus Sternberg,* although he has not definitely determined the bacterium of yellow fever, has made out that it does not liquefy gelatine when access of air is permitted. Certain varieties, as the bacillus fluorescens, have a peculiar color reaction, others produce nodules, thick or delicate cloud-like masses, spicular or branched growths, and some few have a characteristic odor.

The behavior of the bacteria in relation to certain chemical reagents is frequently very characteristic. Bujwid† asserts that if to a ten hour old culture of Koch's comma bacillus five or ten per cent. hydrochloric, nitric or sulphuric acid be added, a rose-violet color is produced, which generally grows more intense in the course of half an hour. This "cholera-red" is, so far as I am aware, unique.

The reactions with aniline dyes is of more practical importance. As a rule the mineral acids decolorize objects stained with these dyes, but it is possible to fix the stains in certain bacteria by mordants of different kinds, so that everything else in the specimen will be decolorized but the special bacterium. Erlich's familiar stain for tubercle is an instance of this. He uses a solution of aniline oil in water as a mordant for gentian violet, and on decolorizing with twenty-five per cent. nitric acid, the tubercle bacilli, if present, will retain their stain, appearing deeply colored on a colorless ground." Of course it is then possible to bring them into greater prominence by staining the ground with a deeply contrasting color.

Aside from the reaction of the bacteria with staining reagents, their behavior in regard to oxygen is of importance. The bacillus of tetanus grows in an atmosphere devoid of oxygen, and

*Sternberg. Report on Etiology, etc. of Yellow Fever, p. 117.

†Report on Cholera in Europe and India. E. O. Shakespeare, p. 641. See also Brieger in Deutsche Med. Woch., 1887, p. 303.

pure cultures may be and are best grown in hydrogen or nitrogen. This bears out our clinical experience, by which we see that a deep punctured wound is more apt to be followed by tetanus than an open one, other things being equal. The vibrios of butyric fermentation and the putrefactive bacteria show a marked difference in behavior according to the amount of air present; if limited, fermentation; if abundant, putrefaction occurring. So invariable is this law, that Pasteur describes fermentation as "Life without air."

The final and conclusive test by which species of bacteria may be distinguished is actual experiment on a living animal. As this is a test of the pathogenic nature of these minute beings, it will be referred to again in another connection.

By judicious use of the various methods indicated above the micro-organisms may be classified, and then the problem of their relation to disease confronts us. To be regarded as the cause of a disease the suspected bacterium must fulfil the following conditions:

1. It must be present in all cases of the disease.

2. It must not be found in the body except during the course of the disease.

3. It must not produce the disease when properly introduced into an animal susceptible to that particular disease.

The second of these three conditions must be interpreted with liberality, for, as was shown above, there are conditions under which the same bacterium seems to be malignant at one time and not at another. It is therefore possible for a micro-organism to be in the system and yet not produce its specific effect.

It is not my purpose to go deeply into the varieties of pathogenic bacteria. A very few, and those few the ones of greatest practical importance, will suffice for my purpose in this paper, for I merely wish to show the manner in which these minute creatures produce specific diseases.

The most brilliant success that bacteriology has achieved, from a practical point of view, has been in the treatment of wounds. Tyndall made a number of experiments to show that the motes in the air were the origins of the micro-organisms found in putrescent material, the result of which experiments can best be summed up in his own words.

"Build a little chamber, and provide it with a door, windows, and window shutters; let an aperture be made in one of the shutters through which a sunbeam can pass; close the door and shutters so that no light shall enter save through this aperture; the track of the sunbeam is at first perfectly plain and vivid in the air of the room. If all disturbance of the air be avoided, the luminous track will become fainter and fainter, until at last it disappears absolutely, and no trace of the beam is to be seen. What rendered the beam visible at first? The floating dust of the air, which, when thus illuminated and observed, is as palpable to sense as dust or powder placed on the palm of the hand. In the still air the dust gradually sinks to the floor or sticks to the walls or ceiling, until finally, by this self-cleansing process, the air is entirely freed from mechanically suspended matter. Thus far, I think, we have made one footing sure. Let us proceed. Chop up a beefsteak, and allow it to remain for two or three hours just covered with warm water; you thus extract the juice of the meat in its concentrated form. By properly boiling the liquid and filtering it, you soon obtain from it a perfectly transparent beef tea. Expose a number of vessels containing this tea to the moteless air of the chamber, and a similar number to dust-laden air. In three days every one of the latter stinks, and with the microscope every one of them is found swarming with the bacteria of putrefaction. After three months or three years, the beef-tea within the chamber, if properly sterilized in the first instance, will be found as sweet and clear as it was the first moment it was put in. There is absolutely no difference between the air within and the air without, save that the one is dustless and the other dust-laden. Clinch the experiment thus: Open the door of the chamber and allow the air to enter it. In three days afterwards you have every vessel within the chamber swarming with bacteria in a state of active putrefaction. Multiply your proof by building fifty chambers instead of one; employ every imaginable infusion of flesh, fish, fowl and viscera, and of vegetables of various kinds; if, in all these cases, you find the dust invariably producing its crop of bacteria, while neither the dustless air nor the nutritive infusion, nor both together, are ever able to produce this crop, your conclusion is simply irresistible that the dust of the air contains the germ of the crop which has appeared in your infusion. I repeat that there is no inference in experimental science more certain than this one."

Acting on these facts, and the results of Pasteur's experiments in the same direction, Lister conceived the idea that by excluding these germs from a wound, or killing them should they gain admittance, that putrefactive processes would not occur in the wound, and that healing by "first intention" would occur, and so he devoted much care and study to dressings to accomplish this purpose. At first shellac and carbolized putty were used, and a spray employed to sterilize the atmosphere in the neighborhood of the wound, but experience has shown that much of the original dressing was unnecessary, and at present the dressings are much simpler, though equally effective.

The principal opponent of antisepsis is Mr. Lawton Tait. He points with just pride to results which he asserts were obtained without antisepsis, but so thorough is his asepsis, that antisepsis is unnecessary. If no micro-organisms be admitted, it is obviously useless to take measures to kill them. He uses boiling water to sterilize his instruments, sponges and dressings, and boiled water for irrigating the field of operation. Heat is one of the best of germicides, and for one using heat as a sterilizer to deride antisepsis and claim not to practice it, is remarkable. He prefers heat to chemical agents as a germicide—that is all. Would a surgeon using chloroform instead of ether be justified in saying that he did not use anæsthesia? Yet this is Tait's position.

Anthrax, or malignant pustule, is a disease usually originating among cattle, and from them communicated to man. It particularly attacks wool-sorters, and usually ends in death from the many abscesses incident to the disease. The cause of this serious disorder has been definitely determined to be a bacillus found in great numbers in the blood and tissues of those affected. This bacillus is 5-10 micromillimetres long and 1-1.25 mm. broad. It is of great interest from a purely scientific point of view, as it illustrates many phenomena in relation to these forms of life. If the bacilli be cultivated in a liquid medium, reproduction takes place by the formation of spores, which are seen as bright oval spots marking the direction of the rods. Upon solid culture media the rods segment transversely before giving rise to the spores, the cell-membrane of each section becoming the membrane of the spore, but having a small lump of protoplasm at each end, that can be stained. The precise significance of this variation is unknown.

But a still more interesting fact is that the culture media have the power of increasing or diminishing the virulence of the bacilli to a great extent. If one-third per cent. of lactic acid be added to the cultures, the virulence of the bacteria is increased two-fold, whereas cultures kept at a temperature of 107° to 109° F. for three or four weeks are greatly attenuated, and a temperature of 131° F. deprives the bacilli of their virulence, though immunity may be obtained by inoculation with them. It is also found that attenuation may be obtained by passing the bacilli through various animals. Thus if a rabbit be inoculated from a fresh culture, a guinea-pig from the rabbit, a goat from the guinea-pig and so on, the bacilli become so altered as to become incapable of transmitting the disease, or, at least, will only transmit a mild variety of it. There is evidently some analogy between this attenuation and the influence of diet alluded to above.

In regard to the mode in which the bacilli of anthrax cause death, the most plausible view, and that best supported by experimental evidence, is that advanced by Hoffa in *Die Natur des Miltzbrandgiftes*, published at Wiesbaden in 1886. This is, in brief, that the bacilli separate the complex combinations in the organism into toxic substances. He made artificial cultures with the greatest care, on sterilized meat kept for several weeks at a temperature of 98.6° F., and attenuated the product by several methods. This attenuated product he used experimentally on frogs, mice, guinea-pigs and rabbits, and produced symptoms of intoxication. At first, after the inoculation, increased action of the heart and acceleration of breathing was observed, then the animals became somnolent, respiration deep, slow and irregular, pupils dilated, temperature subnormal, diarrhœa with bloody feces and speedy death. At the necropsy, the heart was contracted, blood dark colored, and ecchymoses found in the pericardium and peritoneum. No such results follow the injection of sterilized meat-juice, and the deduction was inevitable that some ptomaïne elaborated by the bacilli was the active agent.

The time at my disposal forbids me to go further into the discussion of special bacteria and their specific effects, but before closing I wish to speak of the bacillus of typhoid fever. I have done some experimental work with it during the past summer, but as yet cannot say that I have accomplished anything very definite. My cultures were made from the feces of a typhoid patient, the colonies of bacilli typhosi being isolated as well as I could isolate them by roll-cultures. I was able to produce profuse diarrhœa, and at the necropsy found the lymphatic glands swollen, but that is all. Whether the animals used (mice) are capable of taking the disease, I do not know; I can but state the facts as I found them.

